# Improving C_4_ photosynthesis to increase productivity under optimal and suboptimal conditions

**DOI:** 10.1093/jxb/erab327

**Published:** 2021-07-16

**Authors:** Cristina R G Sales, Yu Wang, Jochem B Evers, Johannes Kromdijk

**Affiliations:** 1 Department of Plant Sciences, University of Cambridge, Downing Street, Cambridge CB2 3EA, UK; 2 Institute for Genomic Biology, University of Illinois at Urbana-Champaign, Urbana, IL, USA; 3 Centre for Crops Systems Analysis (WUR), Wageningen University, Wageningen, The Netherlands

**Keywords:** Agriculture, C_4_ pathway, crop yield, modelling, NADP-ME, photosynthesis, Rubisco, source–sink

## Abstract

Although improving photosynthetic efficiency is widely recognized as an underutilized strategy to increase crop yields, research in this area is strongly biased towards species with C_3_ photosynthesis relative to C_4_ species. Here, we outline potential strategies for improving C_4_ photosynthesis to increase yields in crops by reviewing the major bottlenecks limiting the C_4_ NADP-malic enzyme pathway under optimal and suboptimal conditions. Recent experimental results demonstrate that steady-state C_4_ photosynthesis under non-stressed conditions can be enhanced by increasing Rubisco content or electron transport capacity, both of which may also stimulate CO_2_ assimilation at supraoptimal temperatures. Several additional putative bottlenecks for photosynthetic performance under drought, heat, or chilling stress or during photosynthetic induction await further experimental verification. Based on source–sink interactions in maize, sugarcane, and sorghum, alleviating these photosynthetic bottlenecks during establishment and growth of the harvestable parts are likely to improve yield. The expected benefits are also shown to be augmented by the increasing trend in planting density, which increases the impact of photosynthetic source limitation on crop yields.

## Teaser

We review the case for improving photosynthesis to increase yields in C_4_ crops, and highlight potential strategies to alleviate bottlenecks limiting C_4_ photosynthetic performance under optimal and suboptimal conditions.

## Introduction

The global human population has seen a staggering increase over the last century, and is currently estimated to be 7.8 billion people (United Nations, 2019). This signifies a tripling of the population compared with 2.6 billion people in 1950 and, although this explosive growth is projected to gradually taper off, substantial further population growth is still predicted (UN Population Division projections). Current projections suggest that by 2050, the global population will have grown to 9.6 billion. Providing enough food for all these additional mouths will be challenging indeed. Historically, human population growth has often been a concern, as exemplified by Thomas Malthus’ two-century-old statement that ‘Population will always grow more rapidly than food supplies until numbers are reduced by war, disease or famine’ (Malthus, 1798). Malthus’ prediction has often been used to emphasize the power of science and technology, allowing human society to outpace his doomsday scenario. Indeed, most commodity crop yields have shown steady increases for many decades (e.g. Ray *et al.*, 2013), and further increases may well be possible via improvements in farm management, plant breeding, as well as via utilization of transgenic (e.g. Pellegrino *et al.*, 2018) and gene editing technologies. Improvements in crop yields can be achieved via development of new higher yielding varieties or via closure of the so-called ‘yield-gap’ between attainable and realized yield (Foley *et al.*, 2011). With regards to the former, the plant breeding strategies of the green revolution have led to spectacular improvements in yield potential via increases in yield components such as harvest index and light capture efficiency, but less so for the conversion efficiency of captured solar energy to energy contained in plant biomass. Theoretical analyses of the maximum conversion efficiency (Zhu *et al.*, 2010) have provided estimates for an upper limit of 4.6–6%. From the few existing measurements of this conversion efficiency in farmers’ fields (reviewed in Zhu *et al.*, 2010), even the most productive crop canopies achieve less than one-third of the computed upper limit, mostly due to losses in photosynthetic efficiency. Thus, improving photosynthetic efficiency may have potential to increase crop yields, as demonstrated by a range of proof-of-concept studies for this strategy in C_3_ species (reviewed by Simkin *et al.*, 2019).

## Increasing crop productivity via improving photosynthetic efficiency: a C_3_ story?

**Fig. 1.**
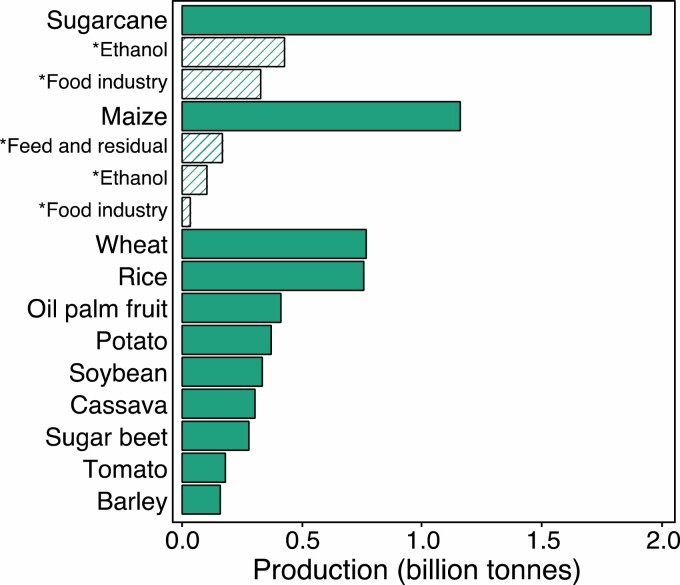
Annual global production of the 11 crops highest in production quantity. The C_4_ NADP-ME sugarcane and maize are at the top of the ranking. Data shown by the solid bars were extracted from the Food Agriculture Organization database (FAO, 2020). *Patterned bars show the approximate amount of different uses of the total production of sugarcane in Brazil (CONAB, 2018) and of maize in the USA (USDA, 2020a).

**Fig. 2.**
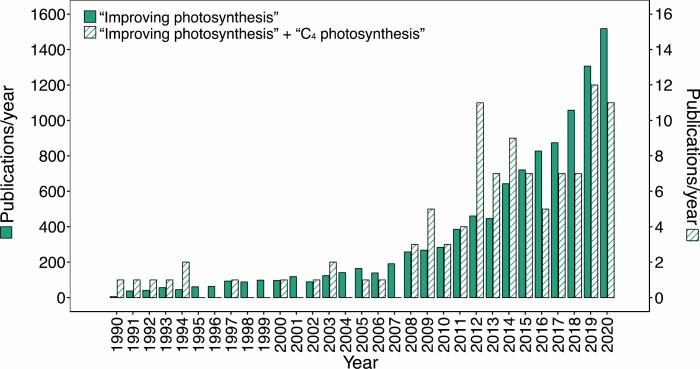
Number of publications per year in subject area ‘Plant Sciences’. The topic ‘Improving photosynthesis’ is shown by the solid green bars, and the number of publications per year ‘C_4_ photosynthesis’ found within this group is shown by the patterned bars. Search results were generated using Web of Science on 19 February 2021.

The vast majority of studies looking at photosynthetic efficiency gains to improve crop yield focus on species with C_3_ photosynthesis. In contrast to C_3_ photosynthesis, species with C_4_ photosynthesis drive a biochemical CO_2_-concentrating mechanism (CCM), which enhances the operating efficiency of Rubisco and competitively inhibits ribulose bisphosphate (RuBP) oxygenation and associated photorespiratory losses (Hatch, 1971; Dai *et al.*, 1993). The CCM also allows plants to function with limited stomatal opening, which reduces water loss through transpiration and consequently increases photosynthetic water-use efficiency (WUE). In addition, less Rubisco is needed, which accounts for most of the nitrogen invested in leaves, and thus increases nitrogen-use efficiency (Ghannoum *et al.*, 2005). Although only ~3% of plant species use the C_4_ pathway (Sage *et al.*, 2012), C_4_ species are strongly over-represented in our agricultural crops, and their importance in supplying food, feed, and fuel is hard to overstate (USDA, 2020a; Fig. 1). As C_4_ photosynthetic species are topping the list of the most highly produced commodities (USDA, 2020a; Fig. 1), novel strategies to improve their productivity should be highly impactful. Despite this, analysis of the research output of the last three decades suggests that only ~1% of research on improving photosynthetic efficiency is focusing on C_4_ photosynthesis (Fig. 2); and even this might be an overestimate, considering that out of the 104 references found with this search, 14 studies are focusing on C_4_ photosynthesis as a means to improve photosynthetic efficiency in C_3_ species.

So why is there less focus on improving the efficiency of C_4_ photosynthesis? The attributes of C_4_ photosynthesis provide an advantage over C_3_ photosynthesis at high light and high temperatures (Sage and Zhu, 2011) and theoretically even at mildly chilling temperatures (Long and Spence, 2013). Does this mean that the conversion efficiency of solar energy to biomass is closer to its biological limit in C_4_ species? Based on the aforementioned theoretical analysis of potential conversion efficiency (Zhu *et al.*, 2010), C_4_ species have an intrinsic advantage compared with C_3_ species but still fall short of the theoretical upper limit by a considerable margin. Thus, the case for improving photosynthetic efficiency to increase crop yield may also hold promise for C_4_ photosynthesis (see also von Caemmerer and Furbank, 2016). Here we therefore review potential strategies for improving C_4_ photosynthesis under optimal and suboptimal conditions.

## How could C_4_ photosynthesis be improved under non-stressed conditions?

**Fig. 3.**
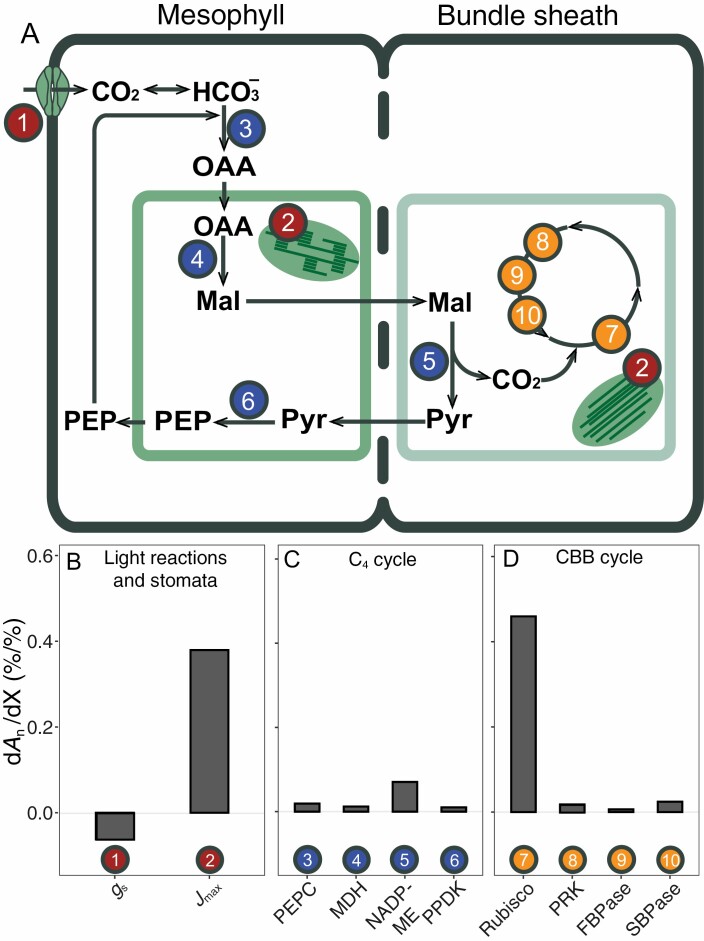
Simulated control coefficients of individual factors over net CO_2_ assimilation rate (*A*_n_) during steady-state conditions. Light reactions and stomatal diffusion (B), C_4_ (C) and CBB cycle (D) enzymes, in maize. The individual factors are numbered and indicated in the diagrammatic representation in (A). Control coefficients were computed as the first derivative of *A*_n_ normalized to each respective control factor X (d*A*_n_/dX). Environmental settings for the simulation were air temperature of 28 °C, photosynthetically active radiation of 1800 μmol m^−2^ s^−1^, ambient CO_2_ concentration of 410 ppm, and air vapour pressure deficit (VPD) of 1.5 kPa. For a full description of the model and parameters, see Wang *et al.* (2021).

**Fig. 4.**
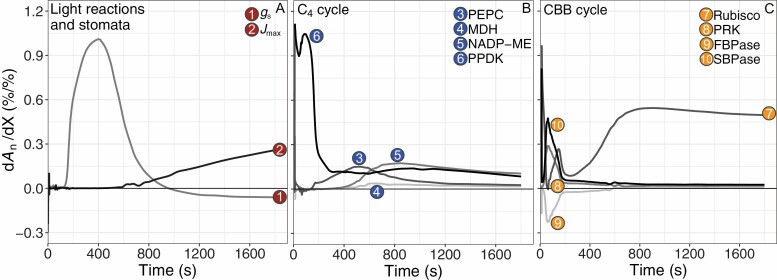
Simulated control coefficients of individual factors over *A*_n_ during a step increase in photosynthetically active radiation. Light reactions and stomatal diffusion (A), C_4_ (B), and CBB cycle (C) enzymes of *A*_n_ in maize. The individual factors are numbered and indicated in the diagrammatic representation in Fig. 3A. Control coefficients were computed as the first derivative of *A*_n_ normalized to each respective control factor X (d*A*_n_/dX). Photosynthetically active radiation was changed from 0 to 1800 μmol m^−2^ s^−1^ at time=0 s. Other environmental settings for the simulation were air temperature of 28 °C, ambient CO_2_ concentration of 410 ppm, and air vapour pressure deficit (VPD) of 1.5 kPa. For a full description of the model and parameters, see Wang *et al.* (2021).

In the following paragraphs, we explore in which ways C_4_ photosynthesis could be improved. Focusing on the NADP-malic enzyme (ME) subtype (Box 1), in this section we will first look at factors that have control over the rate of CO_2_ assimilation under non-stressed conditions. The interdependence of the C_4_ acid shuttle and Calvin–Benson–Bassham (CBB) cycle across two different photosynthetic cell types (mesophyll, M; and bundle sheath, BS) makes it more difficult to pinpoint specific control factors in C_4_ photosynthesis, compared with C_3_ photosynthesis. In addition, the demands for ATP and NADPH in M and BS cells are distinct, and balancing the energy (Bellasio and Griffiths, 2014; Kromdijk *et al.*, 2014) between both compartments is important for efficient functioning of C_4_ photosynthesis. In addition, there is significant carbon exchange between the CBB and C_4_ cycle (Arrivault *et al.*, 2017), which probably helps to maintain flexibility to respond to variable environmental conditions. To account for this complexity, metabolic models which capture the kinetics of all the major reaction and diffusion steps in C_4_ photosynthesis can be used to identify the relative control exerted by any of the modelled factors over the rate of CO_2_ assimilation, by computing control coefficients, defined as the relative change in net CO_2_ assimilation rate (*A*_n_), as a result of a relative change in the control factor. Using their model for NADP-ME photosynthesis (Fig. 3) to simulate the control of individual factors over the rate of assimilation, Wang *et al.* (2021) computed that under high light, control over steady-state *A*_n_ is shared between Rubisco in the CBB cycle (d*A*_n_/dRubisco=0.46) and *J*_max_, namely the capacity for chloroplastic electron transport (d*A*_n_/d*J*_max_=0.38). Using the same model to simulate a step change in light intensity from darkness to 1800 μmol m^−2^ s^−1^ (Fig. 4), a strong transient control of pyruvate-orthophosphate di-kinase (PPDK) during the first minutes was predicted (Fig. 4B), to ramp up metabolic pools in the C_4_ cycle. Similarly, but less pronounced, sedoheptulose-1,7-bisphosphatase (SBPase) and phosphoribulokinase (PRK) share significant transient control, consistent with their role in the regeneration of CBB cycle substrate (Fig. 4C). The concomitant negative transient control of chloroplastic fructose-1.6-bisphosphatase (FBPase) can be explained by the role of fructose-6-phosphate in starch formation (Raines, 2003), which would compete with the availability of substrate in the CBB cycle. After *A*_n_ is induced far enough to significantly deplete intercellular CO_2_ concentrations, the control shifts transiently to stomatal conductance (*g*s) and phosphoenolpyruvate carboxylase (PEPC; Fig. 4A, B), before finally settling on the steady-state control by Rubisco and *J*_max_ shown in Fig. 3.

Box 1. **The NADP-ME C_4_ photosynthesis subtype**

Dual cell-type C_4_ photosynthesis can be classified into three biochemical subtypes, NADP-ME, NAD-ME, and PEPCK, based on the enzyme used to decarboxylate C_4_ acids (Hatch *et al.*, 1975). Here, the main focus is on NADP-ME C_4_ grasses of the Andropogoneae clade which represent some of the most important cultivated plants with a large impact on food, feed, and fuel production (Christin *et al.*, 2009; Fig. 1).

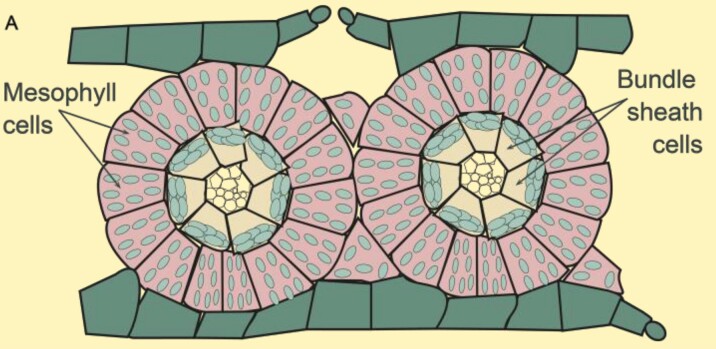

**Chloroplast differentiation in NADP-ME C_4_**

NADP-ME C_4_ grasses show classical ‘Kranz’ leaf anatomy (A). Low PSII abundance in BS thylakoids prevents high rates of whole-chain electron transfer. Instead, reductant is supplied via the malate shuttle, and ΔpH formation and ATP synthesis are predominantly driven by CET (Hatch, 1987; B and C). Chloroplasts in M cells retain the capacity to undergo aggregative movements in response to environmental stresses (Yamada *et al.*, 2009), but in BS cells are more confined to their centrifugal position, which may facilitate metabolite transfer between M and BS (Maai *et al.*, 2011).

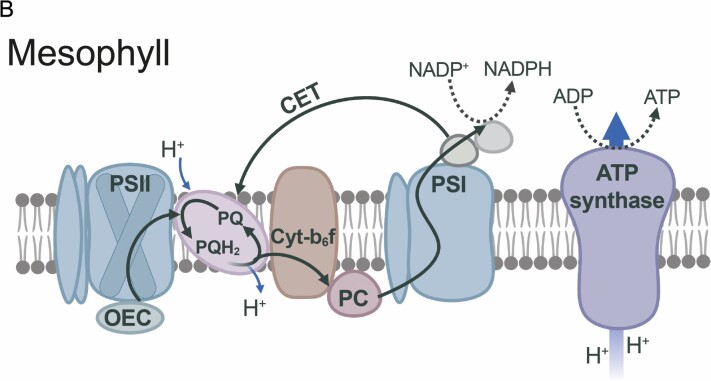

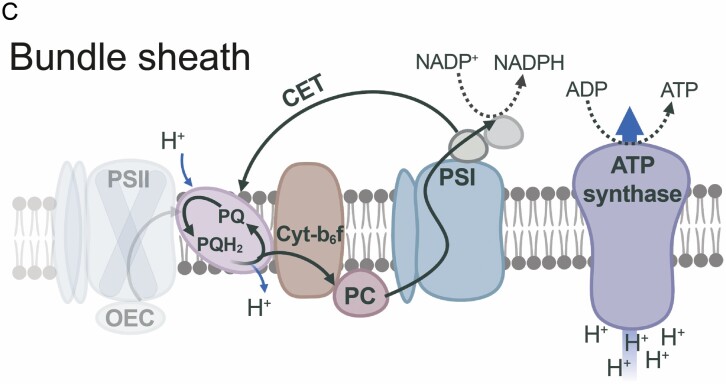

**Flexibility in the decarboxylation pathway**

- Carbon fixation starts in the M cytosol (D). CO_2_ is converted into HCO_3_^−^ by CA and is used in the carboxylation of PEP (catalysed by PEPC) forming the C_4_ organic acid OAA. OAA is converted to malate by MDH in M chloroplasts and diffuses to the BS cells. Decarboxylation of malate by NADP-ME in BS chloroplasts elevates the CO_2_ concentration around Rubisco and provides NADPH.
- Pyruvate diffuses back to the M cells, where PEP is regenerated by PPDK at the expense of 2 ATP/PEP (Kanai and Edwards, 1999).
- Significant activity of PEPCK (dotted arrows) can supplement the NADP-ME route (Furbank, 2011; Yin and Struik, 2021); up to 25% in maize (Hatch, 1971); present in sugarcane (Calsa and Figueira, 2007; Sales *et al.*, 2018; Cacefo *et al.*, 2019); but undetectable in sorghum (Gutierrez *et al.*, 1974).
- Pyruvate can also be transaminated into alanine, before diffusing back to the M cell (Schlüter *et al.*, 2019; faded arrows), which may help to balance nitrogen metabolism (Wang *et al.*, 2014).

Abbreviations: Ala, alanine; Ala-AT, alanine aminotransferase; Asp, aspartate; Asp-AT, aspartate aminotransferase; BS, bundle sheath; CA, carbonic anhydrase; CBB, Calvin–Benson–Bassham cycle; CET, cyclic electron transfer; M, mesophyll; Mal, malate; MDH, malate dehydrogenase; NAD-ME, NAD-malic enzyme; NADP-ME, NADP-malic enzyme; OAA, oxaloacetate; OEC, oxygen-evolving complex; PC, plastocyanin; PEP, phosphoenolpyruvate; PEPC, phosphoenolpyruvate carboxylase; PEPCK, PEP carboxykinase; PPDK, pyruvate-orthophosphate-dikinase; PQ, oxidized plastoquinone; PQH_2_, reduced plastoquinone; Pyr, pyruvate; RuBP, ribulose 1,5 bisphosphate. Schemes B and C were created with BioRender (https://biorender.com/).

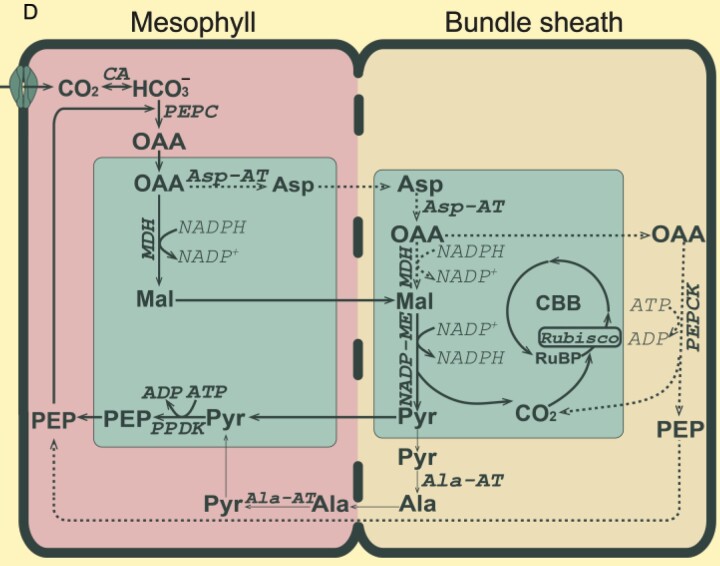

### CBB cycle control over C_4_ photosynthesis

It is well known that the Rubisco catalytic rate is relatively slow, and catalytic improvement may be constrained by a trade-off with its specificity for CO_2_ over O_2_ (Zhu *et al.*, 2004b; Carmo-Silva *et al.*, 2015). Lower abundance of Rubisco in C_4_ plants also affects the levels of CBB metabolites, in particular RuBP, which was significantly lower in four C_4_ species, relative to five C_3_ species (Arrivault *et al.*, 2019). The high CO_2_ environment around Rubisco in C_4_ plants seems to have relaxed the selective pressure for high specificity, resulting in a higher catalytic turnover rate (*k*_cat_) of C_4_ Rubisco compared with C_3_, at the expense of affinity for CO_2_ (*k*_c_) (Kapralov *et al.*, 2011). Substantial diversity in Rubisco activity and catalytic properties among C_4_ species is found (Hermida-Carrera *et al.*, 2016; Orr *et al.*, 2016; Sharwood *et al.*, 2016a, b; Kolbe *et al.*, 2018b), possibly reflecting differences in time since the evolution of the C_4_ pathway between different lineages, as well as systematic differences between different decarboxylation types (Ghannoum *et al.*, 2005). Swapping the nuclear-encoded Rubisco small subunit for transgenic or chimeric versions can be used to alter Rubisco kinetic properties (Ishikawa *et al.*, 2011; Atkinson *et al.*, 2017) and may offer a tractable route to manipulating *k*_cat_.

A different strategy to increase Rubisco capacity is via enhancing its content. Space constraints in the BS chloroplasts were initially thought to limit Rubisco content in C_4_ species (Sage and McKown, 2006). However, recent work shows that C_4_ BS chloroplasts may be able to house Rubisco amounts sufficient to alleviate its control over C_4_ photosynthesis (Pignon *et al.*, 2019). Indeed, using combined overexpression of Rubisco large and small subunits together with the Rubisco assembly chaperone RUBISCO ASSEMBLY FACTOR 1 (RAF1) in maize, Salesse-Smith *et al.* (2018) achieved a >30% increase in Rubisco content. The Rubisco-overexpressing maize plants showed significant increases in CO_2_ assimilation as well as plant growth. However, the gains were limited due to a decline in Rubisco activation state in the overexpression lines. Although Rubisco activase (Rca) content appears typically in excess in C_4_ species (von Caemmerer *et al.*, 2005), it is possible that overexpression of Rca in parallel with increasing Rubisco content will further enhance the potential of this strategy (Salesse-Smith *et al.*, 2018).

### Electron transport capacity control over C_4_ photosynthesis

In C_4_ species, electron transport occurs in both M and BS cells, but the energy supply and demand vary considerably between both photosynthetic cell types. In NADP-ME C_4_ photosynthesis, which is simulated by the model, M cells perform whole-chain electron transport, whereas BS cells have low PSII activity. High rates of cyclic electron transport around PSI in BS chloroplasts drive ATP synthesis (see Box 1), whereas NADPH is instead supplied by the M cells via the malate shuttle. In an attempt to enhance cyclic electron transport in NADP-ME-type C_4_ plants, Tazoe *et al.* (2020) overexpressed PGR5 in *Flaveria bidentis*, an NADP-ME C_4_ dicot. Although this led to a higher electron sink downstream of PSI and alleviated acceptor-side limitation of PSI under fluctuating light, it did not impact CO_2_ assimilation.

Cyclic and linear electron flow are both subject to so-called ‘photosynthetic control’ via the cytochrome *b*_6_*f* (Cyt-*b*_6_*f*) complex, which upon acidification of the thylakoid lumen causes deceleration of the oxidation of plastoquinol and limits electron flow towards plastocyanin (Foyer *et al.*, 2012). A decrease in Cyt-*b*_6_*f* content and proportional decline in electron transfer rates was observed in antisense Rieske FeS mutants of the C_3_ species tobacco (Price *et al.*, 1995). More recently, the opposite strategy, overexpression of Rieske FeS, was shown to be sufficient to elevate Cyt-*b*_6_*f* content in the NADP-ME model C_4_ grass *Setaria viridis* (Ermakova *et al.*, 2019). The resulting increase in *J*_max_ also improved the rate of CO_2_ assimilation, via enhanced quantum yields of both photosystems and a decrease in loss of energy via non-photochemical quenching (NPQ). Slow temporal kinetics of NPQ have also been predicted to decrease CO_2_ fixation (Murchie and Niyogi, 2011), and faster NPQ in transgenic tobacco plants was shown to significantly boost photosynthetic efficiency and productivity (Kromdijk *et al.*, 2016). Model simulations using maize PSII quantum yield recovery kinetics predict an even greater potential impact than found in tobacco (Zhu *et al.*, 2004a), suggesting that the transgenic strategy by Kromdijk *et al.* (2016) may also have merit in C_4_ species.

### C_4_ cycle control over C_4_ photosynthesis rates

None of the C_4_ cycle enzymes individually seems to have strong control (<0.07 d*A*_n_/dX in the model calculations) over the rate of photosynthesis under steady-state, highlight conditions (Fig. 3C), which is consistent with the notion that the CCM has to operate at slightly higher rates than the CBB cycle in order to achieve the CO_2_-concentrating effects while accounting for overcycling due to retrodiffusion of CO_2_ (termed leakiness, Farquhar, 1983; reviewed by Kromdijk *et al.*, 2014). That is, when *A*_n_ is already saturated with CO_2_, any further increases in CO_2_ concentration should not affect the rate of photosynthesis and instead increase leakiness and concomitant energy loss. In addition, the C_4_ cycle rate also determines the rate of reductant shuttle from the M to BS chloroplasts. Although C_4_ acid transporters may also play a significant role (e.g. Weissmann *et al.*, 2016), the C_4_ cycle rate is primarily controlled by the activity of NADP-malate dehydrogenase (NADP-MDH), the only thioredoxin-regulated enzyme in the C_4_ cycle (Leegood and Walker, 1999). However, activity of NADP-MDH under steady-state highlight conditions is typically in excess of the net assimilation rate (Usuda *et al.*, 1984). Activity of NADP-MDH in mutant lines of *F. bidentis* could be reduced to <50% before CO_2_ fixation capacity was affected (Trevanion *et al.*, 1997), which led the authors to suggest that instead of a direct regulatory role in photosynthesis, the covalent regulation of NADP-MDH activity may function to keep the chloroplastic NADP pool largely reduced and to limit reductant transfer from the chloroplast under darkness.

In contrast to steady-state, under transient conditions strong control coefficients are predicted for C_4_ cycle enzymes. During photosynthetic induction due to a change in light intensity from 0 to 1800 μmol m^−2^ s^−1^, PPDK exerts a strong control over *A*_n_ during the first minutes (Fig. 4). PPDK is an important enzyme in C_4_ photosynthesis, controlling the regeneration of PEP, the substrate for the primary carboxylation event (Chastain *et al.*, 2011). The importance of PPDK for non-steady state photosynthesis is well known (Usuda *et al.*, 1984). However, PPDK can be rapidly activated via dephosphorylation in response to changes in light intensity (Chen *et al.*, 2014). Consequently, in response to a step increase in light, PPDK increases activity much faster than photosynthesis, which is consistent with the notion that the transient control coefficient of PPDK (Fig. 4B) is not associated with direct control over the carbon uptake flux, but rather with building up the large metabolic pools essential for C_4_ photosynthesis (Stitt and Zhu, 2014). PPDK corresponds to 7–10% of the protein content of M cells (Edwards *et al.*, 1985), and its activity was found to exceed the rate of photosynthesis only slightly in maize (Usuda *et al.*, 1984). In addition, small reductions in PPDK gene expression and amounts led to lower assimilation rates in *F. bidentis* (Trevanion *et al.*, 1999). However, since a significant fraction of PPDK (up to one-third) remains phosphorylated under fully activated conditions (Edwards *et al.*, 1985), some overcapacity seems to exist.

As mentioned above, after *A*_n_ is induced far enough to significantly deplete *C*_i_, the control over *A*_n_ shifts strongly to stomatal conductance and to some extent to PEPC. Extractable activity of PEPC is typically several-fold higher than *in vivo* activity (Laisk and Edwards, 1997) due to strong post-translational control by metabolites as well as reversible phosphorylation. Reversible phosphorylation of a PEPC serine residue increases its activity, while at the same time it reduces the inhibitory effect of malate and aspartate and increases the sensitivity to activation by sugar-phosphates (and glycine in monocots) (Doncaster and Leegood, 1987; Leegood and Walker, 1999; Gowik and Westhoff, 2011). Structural analysis of the homotetrameric PEPC showed that each monomer contains separate binding sites for the substrate PEP and the allosteric inhibitors malate and aspartate (Schlieper *et al.*, 2014). The evolutionary co-opting of PEPC in C_4_ photosynthesis has led to different kinetic properties in the C_4_ isoform (Gowik and Westhoff, 2011) and several residues responsible for these changes have been discovered. Comparative analysis of crystal structures of C_3_ and C_4_ PEPC identified an arginine to glycine mutation in the C_4_ variant, leading to decreased inhibition by malate/aspartate (Paulus *et al.*, 2013). Another single serine to alanine substitution decreases PEPC affinity for bicarbonate in the C_4_ isoform (DiMario and Cousins, 2019). Based on the balance between *in vivo* PEPC and Rubisco capacity in 49 C_4_ species (Pignon and Long, 2020), it could be hypothesized that a strategy shifting leaf nitrogen (N) investment away from PEPC towards *J*_max_ and Rubisco could be effective to achieve higher rates of photosynthesis. A similar hypothesis was proposed by Kromdijk and Long (2016) for C_3_ species, suggesting that leaf N investment for high *A*_n_ could be more effective by a shift from carboxylation towards regeneration capacity in the CBB cycle. However, considering the non-negligible control of PEPC over non-steady-state photosynthesis (Fig. 4), and the perceived importance of dynamic photosynthesis over total canopy CO_2_ fixation (Murchie *et al.*, 2018; Slattery *et al.*, 2018), there could be significant drawbacks to this strategy.

## How can C_4_ photosynthesis under suboptimal conditions be improved?

**Fig. 5.**
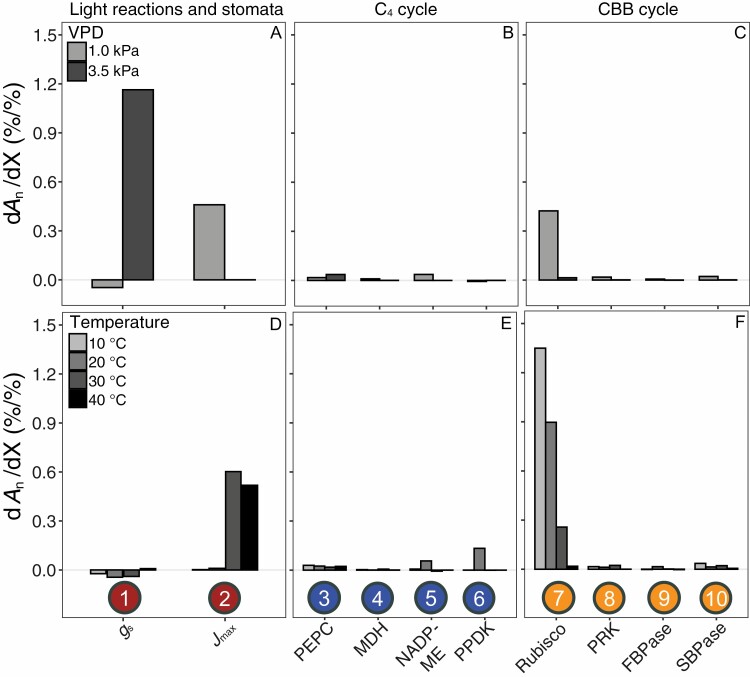
Simulated control coefficients of individual factors over *A*_n_ during steady-state under environmental stress conditions. Light reactions and stomatal diffusion (A, D), C_4_ (B, E), and CBB cycle (C, F) enzymes, during steady-state *A*_n_ in maize under contrasting air vapour pressure deficit (VPD; A–C) and air temperature (D–F). The individual factors are numbered and indicated in the diagrammatic representation in Fig. 3A. Control coefficients were computed as the first derivative of *A*_n_ normalized to each respective control factor X (d*A*_n_/dX). Environmental settings for the simulation were air temperature of 10–40 °C, photosynthetically active radiation of 1800 μmol m^−2^ s^−1^, ambient CO_2_ concentration of 410 ppm, and VPD of 1 or 3.5 kPa. For a full description of the model and parameters, see Wang *et al.* (2021).

Whereas the factors above are important for C_4_ performance under optimal non-stressed conditions, factors controlling photosynthetic rates under suboptimal, stressed conditions are arguably more important for crop productivity. We used the model flux analysis by Wang *et al.* (2021) to account for short-term environmental stresses (Fig. 5) simulating drought [vapour pressure deficit (VPD) changed from 1.5 kPa to 3.5 kPa], heat, and cold stress (air temperature changed between 10 °C and 40 °C). The simulated drought conditions induce a shift from enzymatic factors to diffusional control of *A*_n_ by stomatal conductance (Fig 5A–C), which suggests that under these dry conditions, *A*_n_ becomes limited by CO_2_ supply despite the concentrating action of the C_4_ cycle. The shifts in the control coefficients in response to air temperature (Fig. 5D–F) are reflecting the differential responses of enzymatic activities which are strongly impacted by temperature, and the photosynthetic light reactions, which are much less affected. As a result, low temperatures dramatically increase the control coefficient of Rubisco, whereas under high temperatures, *A*_n_ is primarily controlled by electron transfer capacity. Notably, the co-limitation between Rubisco and *J*_max_ at 30 °C is replaced at 20 °C by co-limitation between Rubisco and C_4_ cycle activity via PEPC, NADP-ME, and PPDK activities.

### Chilling temperature effects on C_4_ photosynthesis

Due to their evolutionary origins from tropical and subtropical regions, most C_4_ species are maladapted to chilling temperatures, in particular in combination with exposure to light which gives rise to chilling-induced photoinhibition (Taylor and Craig, 1971; Long *et al.*, 1983). The most extreme crop example is probably sugarcane, which is particularly chilling sensitive (Grantz, 1989; Głowacka *et al.*, 2016), and severely limited in its latitudinal range. However, even though maize has better chilling tolerance, it is still the most susceptible crop to chilling-induced photoinhibition amongst those grown in temperate regions (Hetherington *et al.*, 1989). Improving resilience to low-temperature conditions in C_4_ crops could have a strong economic impact by increasing latitudinal range, helping to reduce year by year yield variability and decreasing early season competition with weeds. The model predictions suggest that increasing Rubisco activity is key to maintaining photosynthetic capacity under chilling conditions (Fig. 5F). However, transgenic maize lines with increased Rubisco content did not show better performance during chilling conditions, which suggests that Rubisco is not the primary limiting factor that causes maize susceptibility to low temperatures, although Rubisco overexpression may help plants recover faster from chilling events (Salesse-Smith *et al.*, 2020).

Protein lability under low temperatures could play an important role in chilling tolerance. Several important proteins, most notably PEPC (Kingston-Smith *et al.*, 1997; Chinthapalli *et al.*, 2003), PPDK (Du *et al.*, 1999), as well as Rubisco (Kingston-Smith *et al.*, 1997; Du *et al.*, 1999; Pittermann and Sage, 2001*b*; Chinthapalli *et al.*, 2003), show increased rates of breakdown under chilling conditions. It is worth noting that the model calculations shown in Fig. 5 do not consider any effects of protein breakdown. In other words, the enhanced control coefficient which the model predicts for Rubisco activity under chilling temperatures is based purely on the kinetic properties of the enzyme, but in reality it will be compounded by the loss of activity due to protein disintegration. The effects of protein breakdown enhance the control of the cold-labile proteins PEPC and especially PPDK over *A*_n_ under cool conditions. Indeed, comparisons between maize and its chilling-tolerant distant relative *Miscanthus×giganteus* demonstrate that the latter responds to chilling with a strong transcriptional up-regulation of gene expression networks involved in photosynthesis and carbon assimilation, as well as protein synthesis and degradation (Spence *et al.*, 2014) to counteract the enhanced breakdown of proteins such as Rubisco, PEPC, and PPDK (Naidu *et al.*, 2003; Wang *et al.*, 2008; Serrano-Romero and Cousins, 2020), resulting in superior productivity under temperate climates (Dohleman and Long, 2009).

Experiments with chilling temperatures in the presence and absence of illumination show that light, although not a prerequisite for photoinhibition (Ortiz-Lopez *et al.*, 1990), clearly has an exacerbating role in the extent of chilling damage in maize (Taylor and Craig 1971; Long *et al.*, 1983). This is due to the fact that light-harvesting and electron transfer reactions are much less perturbed by low temperature than downstream electron sinks. The resulting imbalance increases the probability of formation of reactive oxygen species (ROS), which are the primary cause of photodamage. Photoprotective and ROS-scavenging mechanisms are therefore important to maintain efficient C_4_ photosynthesis under suboptimal temperatures. The differences between chloroplast populations in BS and M cells in NADP-ME C_4_ species (see Box 1) may need to be considered in this regard. In maize, BS and M cells have distinct antioxidant capacities (Doulis *et al.*, 1997) relative to the availability of reducing power in each compartment (reviewed in Turkan *et al.*, 2018), with BS cells often lacking antioxidant capacity. Under optimal conditions, intracellular transport of reduced and oxidized forms of antioxidants allows continued ROS scavenging in BS cells. However, under low temperatures, the transport between compartments may become impaired, exposing the BS cells to oxidative stress (Kingston-Smith and Foyer, 2000). In addition, although most work on chilling-induced photoinhibition has focused on PSII inhibition, PSI inhibition is particularly prominent under cool conditions combined with fluctuating light (Kono *et al.*, 2014). PSI is especially sensitive to inhibition when PSI electron acceptors are limited and the lack of an efficient repair cycle leads to prolonged recovery times which can span several days (Sonoike, 2011). These characteristics are consistent with the hallmark signs of chilling-induced photoinhibition in maize and sugarcane (Pimentel *et al.*, 2005; Głowacka *et al.*, 2016). Indeed, PSI activity can be strongly reduced by chilling or photoinhibitory treatments (e.g. Baker and Nie, 1994; Savitch *et al.*, 2011; Qiao *et al.*, 2020), which would justify another look at a putative role for PSI in chilling-induced photoinhibition of C_4_ species.

### Drought stress effects on C_4_ photosynthesis

Increasing temperatures and fewer predictable precipitation events caused by global climatic change, are expected to increase the frequency of high VPD conditions and reduced water availability during crop growth (Yuan *et al.*, 2019). High WUE, defined as the amount of carbon fixed per unit of water lost, can help to conserve soil water content for critical moments during the growing season. Although C_4_ species typically have higher WUE than C_3_ plants, the differences diminish under drought conditions (Ripley *et al.*, 2010; Taylor *et al.*, 2010). Under mild drought, a decrease in *C*_i_ can significantly affect saturation levels of CO_2_ around Rubisco. Consequently, *A*_n_ becomes limited by stomatal diffusion (Fig. 5A) as well as by PEPC. In addition, and consistent with the CO_2_ limitation of *A*_n_, work with *Zea mays* lines with strongly reduced carbonic anhydrase (CA) activity suggests that this may also become a minor limitation at low *C*_*i*_ (Studer *et al.*, 2014; Kolbe *et al.*, 2018). Similarly, the leaf internal conductance to CO_2_ from the intercellular airspaces to the sites of fixation (mesophyll conductance, *g*_m_) may also impact *A*_n_ and WUE at low *C*_*i*_ (e.g. Kolbe and Cousins, 2018), but the mechanism and role of *g*_m_ in limiting *A*_n_ in C_4_ species under different environmental conditions are not very well understood. Native responses of PEPC in C_4_ species under water deficit do not show a clear picture, with some results indicating a decrease in activity (Becker and Fock, 1986; Du *et al.*, 1996) and others showing little change or increased activities of PEPC (Saliendra *et al.*, 1996; Foyer *et al.*, 1998; Carmo-Silva *et al.*, 2007; Ghannoum, 2009; Pissolato *et al.*, 2020). Beneficial effects of overexpression of PEPC have been reported for maize plants grown under mild drought conditions, resulting in higher WUE and increased biomass (Jeanneau *et al.*, 2002), but these effects were suggested to stem from a pleiotropic negative effect on stomatal density of the PEPC overexpression, rather than from direct enhancement of the C_4_ cycle. Indeed, transgenic maize with reduced stomatal density (Liu *et al.*, 2015) or increased stomatal sensitivity to abscisic acid (Brugière *et al.*, 2017) had lower *g*_s_ and increased WUE when subjected to drought. In addition, reduced *g*_s_ under high VPD was successfully used as a trait in breeding programmes to produce more drought-tolerant maize (Messina *et al.*, 2020).

After prolonged exposure, drought-induced reductions in *A*_n_ can no longer be rescued by high CO_2_, suggesting that biochemical limitation replaces stomatal conductance as the dominant control factor (Ghannoum, 2009; Ripley *et al.*, 2010; Bellasio *et al.*, 2018). The exact biochemical bottlenecks are not easy to pin-point, but could be related to impaired activities of CBB or C_4_ cycle enzymes. Although effects of drought on different photosynthetic enzymes appear strongly species dependent, impairment of Rubisco under drought conditions is often found (Du *et al.*, 1996; Saliendra *et al.*, 1996; Carmo-Silva *et al.*, 2007; Ghannoum, 2009; Perdomo *et al.*, 2017). However, maize with increased Rubisco content did not have a higher assimilation rate or plant growth under drought stress, and the overexpression was beneficial only for the recovery of photosynthesis after rewatering (Doron *et al.*, 2020). Prolonged drought stress and reduced leaf water content may also negatively affect the integrity of the chloroplastic ATPase, and the resulting decline in ATP synthesis can decrease regeneration of substrates in CBB and C_4_ cycles (reviewed by Ghannoum, 2009). These phenomena are compounded by the enhanced build-up of excess excitation energy under stress conditions, when the absorbed light exceeds the energy requirements to drive C_4_ and CBB cycle activities. Whereas this is true for C_3_ and C_4_ species alike, the capacity for photoprotection under stressed conditions may be impaired in C_4_ plants in a cell type-specific manner. BS chloroplasts appear to have only limited capacity to undergo photoprotective movements. In addition, photorespiration is an important alternative electron sink to dissipate excessive excitation energy in C_3_ plants (Takahashi *et al.*, 2007) which may be impacted by the C_4_ pathway. Photorespiratory mutants are lethal in maize (Zelitch *et al.*, 2009), demonstrating the required presence of the pathway; however, it seems plausible that the capacity of the photorespiratory pathway in C_4_ species may be less sufficient to offer photoprotection under drought and high light stress.

### Heat stress effects on C_4_ photosynthesis

Consistent with the model simulation (Fig. 5), *J*_max_ has been shown to limit C_4_ photosynthesis at superoptimal temperatures (Pittermann and Sage, 2001*a*; Kubien *et al.*, 2003; Dwyer *et al.*, 2007), but the differences in electron transport characteristics between M and BS chloroplasts (Box 1) make it difficult to discern more specific bottlenecks from these data. Additional limitations to C_4_ photosynthesis associated with high temperatures include reduction in Rubisco capacity, RuBP regeneration, and reductions in Rca activity (Crafts-Brandner and Salvucci, 2002; Kubien *et al.*, 2003). Impairment of Rca activity can severely reduce photosynthesis at high temperature (Salvucci *et al.*, 2001; Carmo-Silva and Salvucci, 2012; Perdomo *et al.*, 2017; Scafaro *et al.*, 2018; Degen *et al.*, 2021). Although reduced transcript levels of Rca at high temperature in the C_4_ species *F. bidentis* appeared not to be directly related to Rca protein accumulation (Hendrickson *et al.*, 2008), intrinsic heat sensitivity of Rca in maize did explain decreased Rubisco activation (Perdomo *et al.*, 2017). Differential expression of Rca-α and Rca-β isoforms in response to temperature may contribute to heat tolerance. Rca-α expression in five C_4_ grasses was induced by high temperatures (Kim *et al.*, 2021) and appeared to be involved in Rca hexamer stability. These results are different from what has been seen in the C_3_ grass wheat, in which Rca1β is more thermostable among the three Rca isoforms Rca1β, Rca2β, and Rca1α (Scafaro *et al.*, 2019; Degen *et al.*, 2021), but similar to heat-treated rice (Wang *et al.*, 2010). Specific amino acid substitutions between thermosensitive and thermotolerant isoforms have been identified to act as thermal and regulatory switches in wheat Rca which strongly impact performance under high temperature *in vitro* (Scafaro *et al.*, 2019; Degen *et al.*, 2020). Transgenic expression of the thermostable Rca from *Oryza australiensis* improved yields in heat-stressed *O. sativa* (Scafaro *et al.*, 2018), and a similar strategy based on overexpression of a transgenic or mutated thermally stable Rca isoform may also hold promise for improving photosynthetic heat tolerance in C_4_ species.

## Substantial natural genetic variation in photosynthesis of C_4_ crop species

The C_4_ photosynthetic bottlenecks discussed in the previous paragraphs are summarized in Table 1, which provides a list of key attributes that could have potential to improve photosynthesis. To find out whether these attributes could be improved via breeding, the presence of existing genetic variation in a species germplasm is a prerequisite. Although some diversity has probably been lost during domestication (Doebley *et al.*, 2006), there appears to be significant genetic variation for photosynthetic traits in the germplasm of several C_3_ crops (Driever *et al.*, 2014; Gaju *et al.*, 2016; Carmo-Silva *et al.*, 2017; Pennacchi *et al.*, 2018; Molero *et al.*, 2019; Acevedo-Siaca *et al.*, 2020, 2021), with varying degrees of heritability, in some cases presenting clear opportunities for marker-assisted breeding of future cultivars (e.g. Adachi *et al.*, 2017). In this section, we review progress in using natural genetic variation to improve C_4_ photosynthesis.

**Table 1.** Summary of strategies to improve C_4_ photosynthesis and presence/absence of evidence from simulation modelling (S) or experimental data (E) under non-stressed steady state conditions or photosynthetic induction, cool temperature, high temperature and drought stress

Factor	Strategy for improvement	Non-stressed				Stressed					
		Steady-state		Induction		Cool temperature		High temperature		Drought	
		S (Fig. 3)	E	S (Fig. 4)	E	S (Fig. 5)	E	S (Fig. 5)	E	S (Fig. 5)	E
***g***_**s**_		✘	?	✓	?	✘	?	✘	?	✓	✓_(1)_ ✘_(2)_
**Electron** **transport**	**Enhance CET**		✘_(3)_	✓	?	✘	?	✓	?	✘	?
	**Increase** **Rieske FeS**	✓	✓_(4)_		?		?		?		?
	**Speed up NPQ**		?		?		?		?		?
**C**_**4**_ **cycle**	**PEPC**	✘	✘_(5)_	✓	✓_(5)_	✘	?	✘	?	✘	✓_(6)_
	**NADP-ME**	✘	?	✘	?	✓	?	✘	?	✘	?
	**PPDK**	✘	?	✓	?	✓	?	✘	?	✘	?
**CBB cycle: Rubisco**	**Improve *k***_**cat**_		?		?		?		?		?
	**Change small subunit expression**		?		?		?		?		?
	**Increase content**	✓	✓_(7)_	✓	?	✓	✘_(8)_	✓	?	✘	✘_(9)_
	**Increase Rca** **expression**		?		?		?		?		?

Symbols denote no improvement of CO_2_ assimilation rate (✘), improvement of CO_2_ assimilation rate (✓) or unknown effect (?).

References: 1, Liu *et al.* (2015); 2, Brugière *et al.* (2017); 3, Tazoe *et al.* (2020); 4, Ermakova *et al.* (2019); 5, Laisk and Edwards, 1997; 6, Jeanneau *et al.* (2002); 7, Salesse-Smith *et al.* (2018); 8, Salesse-Smith *et al.* (2020); 9, Doron *et al.* (2020).

### Chilling tolerance

Considerable variation in sensitivity to chilling-induced photoinhibition exists between accessions of different C_4_ species, as shown for example in maize (e.g. Aguilera *et al.*, 1999; Fracheboud *et al.*, 2004; Pimentel *et al.*, 2005) and sorghum (Ortiz *et al.*, 2017), but the mechanistic and genetic basis of this variation still remains largely undefined. The latter study with 304 sorghum accessions showed that there is significant natural variation in the photosynthetic response of sorghum lines to cold stress and their capacity to recover, and several putative genomic regions and candidate genes were identified. The understanding of allelic variants associated with these physiological traits could help identify key processes and genes to manipulate by breeding or engineering approaches (Ortiz *et al.*, 2017). In maize, the replacement of landraces by hybrids has drastically reduced the allelic diversity utilized in elite germplasm. However, large-scale production of doubled-haploid lines from promising landrace accessions coupled with genotyping and broad phenotypic characterization should help to make the allelic diversity of landrace collections more readily available for breeding programmes. This approach was recently applied to European flint maize (Hölker *et al.*, 2019). As an early sign of their potential to improve chilling tolerance in maize, doubled-haploid lines from these European landraces outperformed flint founder lines as well as commercial hybrids in early development across a range of 11 temperate environments.

An alternative strategy to improve chilling tolerance is via introgressions from closely related tolerant C_4_ species. The generation of intergeneric hybrids between chilling-sensitive *Saccharum* and the chilling-tolerant *Miscanthus* which show high levels of chilling tolerance in F_1_ ‘miscanes’ (Kar *et al.*, 2019) can be seen as a first step in this strategy, which could allow production of sugarcane and energy cane cultivars for more temperate climes. The recent publication of the *Miscanthus sinensis* genome (Mitros *et al.*, 2020) will help to accelerate the identification of genomic regions specifically relevant to its superior performance under low temperature.

### Water-use efficiency

C_4_ plants are generally more efficient in water use than C_3_ plants, but might be improved further by leveraging natural variation in WUE traits within crop germplasm (reviewed by Leakey *et al.*, 2019). Leaf-level WUE and its component traits *A*_n_ and *g*_s_ show significant within-species variation in diversity or mapping populations of several C_4_ species, such as switchgrass (Taylor *et al.*, 2016), sorghum (Ferguson *et al.*, 2020a Preprint), and maize (Xie *et al.*, 2020). Sugarcane genotypes with higher WUE, due to lower *g*_s_ in combination with high photosynthetic capacity, were identified by Li *et al.* (2017), which may offer breeding potential for higher WUE. Similarly, Pignon *et al.* (2021) identified significant variation in leaf WUE traits across a collection of contrasting sorghum lines. However, detailed analysis of steady-state and dynamic WUE and its component traits *A*_n_ and *g*_s_ across these accessions also identified inherent trade-offs between trait combinations which may severely limit the potential for improvement. Because *A*_n_ and *g*_s_ are positively correlated, the range of variation in their ratio (WUE) is typically smaller than for the individual component traits. In most of the aforementioned studies, the major source of variation in leaf-level WUE is *g*_s_. Using hierarchical grouping by *g*_s_ classes, Jackson *et al.* (2016) identified leaf intercellular [CO_2_] as a major correlate with leaf-level and whole-plant metrics for WUE in sugarcane. In C_3_ plants, the normalized *C*_i_/*C*_a_ metric and related isotopic proxy (∆ ^13^C) have been successfully applied to develop wheat lines with higher WUE (Condon *et al.*, 2004), which may also be possible in C_4_ species (Ellsworth and Cousins, 2016; Eggels *et al.*, 2021). Δ ^13^C can be strongly genetically determined in C_4_ species such as maize (Gresset *et al.*, 2014; Twohey *et al.*, 2018). However, the theoretical slope of the correlation between ∆ ^13^C and *C*_i_/*C*_a_ in C_4_ species can be negative or positive since the relationship is confounded by the impact of BS leakiness (i.e. the rate of retrodiffusion of CO_2_ from the BS cells relative to the rate of PEP carboxylation). Although BS leakiness is relatively constant under most conditions, it can increase considerably under drought or nutrient stress conditions (reviewed by Kromdijk *et al.*, 2014) and in particular under low light intensity (Kromdijk *et al.*, 2008, 2010). Despite these complications, co-localized quantitative trait loci (QTLs) for ∆ ^13^C and several WUE-related traits have been found in maize (Avramova *et al.*, 2019) as well as in *Setaria* (Ellsworth *et al.*, 2020).

Semi-automated pipelines to characterize leaf-level (e.g. Ferguson *et al.*, 2020a, Xie *et al.*, 2020) or whole-plant WUE (e.g. Feldman *et al.*, 2018) can increase phenotyping throughput dramatically, which helps to alleviate the phenotyping bottleneck. Additional challenges associated with identification of genes or genomic loci underpinning differences in WUE are associated with the polygenic nature of the trait. To improve the reliability of genetic associations, several new approaches are being pioneered. Ferguson *et al.* (2020a) demonstrated that the integration of genome-wide and transcriptome-wide association studies (Kremling *et al.*, 2019) can be used to identify candidate genes for WUE with enhanced confidence. Feldman *et al.* (2018) used their temporally rich dataset of whole-plant WUE in a *Setaria italica×S. viridis* recombinant inbred line population to develop function-valued QTL models based on the average log of the odds score across the time course of the experiment, yielding fewer, higher confidence QTL. Predictive models can also be used to speed up breeding efforts, by linking genomic variation with physiological trait variation, and simulate the impact across a wide range of environments (e.g. Inman-Bamber *et al.*, 2016; Kadam *et al.*, 2019; Wu *et al.*, 2019).

### Heat tolerance

Unfavourably high temperatures can severely impact crop yields. In sugarcane, high temperature induced significant decreases in net assimilation rate, maximal PSII efficiency, and activities of sucrose synthase and sucrose phosphate synthase, all of which were more strongly impacted in heat-sensitive compared with heat-tolerant genotypes (Kohila and Gomathi, 2018). In grain crops, the temperature during reproductive development is particularly critical, and temperatures >30 °C during flowering and seed set can negatively impact yield in maize and sorghum (reviewed by Kaushal *et al.*, 2016). The C_4_ photosynthetic temperature response peaks at a higher temperature than the damage threshold temperature during reproductive development, hence crop breeding programmes have mostly focused on traits associated with reproductive success, such as pollen viability, stigma receptivity, and seed set percentage (e.g. Alam *et al.*, 2017) to improve crop heat tolerance. Substantial variation in heat tolerance is present in germplasm of C_4_ crops such as sorghum (e.g. Chopra *et al.*, 2017) and maize (Cairns *et al.*, 2013; Naveed *et al.*, 2016), but it is often unclear how much of this is underpinned by variation in photosynthetic, rather than reproductive, traits. Using different sowing dates to modulate exposure to moderate seasonal heat stress, Yadav *et al.* (2016) found a significant genotype-specific treatment response in photosynthesis rates across a panel of 21 maize inbred lines, which was correlated with biomass productivity. The genotypic differences in photosynthesis responses were not explained by dark-adapted PSII quantum yields, suggesting that enzymatic inhibition, rather than PSII inactivation, explained the observed differences. Metabolic profiling of two contrasting maize genotypes in response to sudden heat stress identified that the levels of nine key metabolites were strongly predictive for the difference in leaf photosynthetic recovery between the two lines (Qu *et al.*, 2018). If this approach is more generally applicable, it may offer potential for high-throughput screening of photosynthetic heat tolerance. ‘Heat tents’ were used by Sunoj *et al.* (2017) to raise average temperature by 8 °C during growth of a collection of sorghum genotypes, achieving quite severe heat stress, with daytime maximum temperatures kept at 45 °C. The net assimilation rate was decreased in a genotype-dependent manner, and fluorescence-estimated thylakoid damage was weakly correlated with the heat stress inhibition, suggesting that this level of heat stress was sufficient to inactivate PSII. Considering that severe heat stress is particularly damaging to the oxygen-evolving complex of PSII (Murata *et al.*, 2007), chlorophyll fluorescence measurements of PSII efficiency can be used to screen photosynthetic responses to heat stress (Murchie *et al.*, 2018). Ferguson *et al.* (2020b) developed high-throughput screening for heat tolerance in rice by combining visual (stay-green) responses with dark-adapted PSII quantum yields to rapidly increasing temperatures in excised leaf material, which may be applicable to C_4_ species such as sorghum and maize.

Taken together, there appears to be substantial natural genetic variation in photosynthesis within germplasm of C_4_ crops, but physiological interpretation is often too minimal to assess how this variation is connected to the traits summarized in Table 1. Addressing this knowledge gap will require development of high-throughput proxies that specifically inform about the attributes in Table 1.

## How could increased photosynthetic efficiency enhance yield in C_4_ crops?

Whereas the previous paragraphs have reviewed the range of strategies to alleviate photosynthetic bottlenecks in C_4_ crops and the potential to utilize natural genetic variation to make improvements, in this final section, the potential impacts of photosynthetic efficiency gains on yield are reviewed by looking at source–sink interactions in three main C_4_ crops: maize, sugarcane, and sorghum.

### Photosynthetic source activity affects sink establishment and grain filling in major C_4_ crops

The constraints to growth and productivity can be formulated in terms of supply and demand, or source versus sink (for a detailed discussion of the source–sink concept, see White *et al.*, 2016; Chang and Zhu, 2017). Plants need a supply of carbohydrates from photosynthesis, water, and mineral nutrients to provide the building blocks and energetic demands to produce new tissue. When supply is insufficient, the growth that is realized will fall short of the potential growth. If so, growth would be source limited. Alternatively, growth can be constrained by the capacity of the growing parts to accumulate biomass. In this situation, an increase in resource availability would not stimulate growth, which is termed as being sink limited. The relative simplicity of this concept is deceptive, and sink and source limitations are not mutually exclusive, but instead growth patterns continuously reflect a relative balance between source and sink constraints, termed the source–sink balance. The source–sink concept applied specifically to the plant’s carbon economy has been a popular framework for analysis of the interplay between photosynthetic activity and yield. For this purpose, the sink strength of the harvestable parts relative to the photosynthetic activity of the leaves reflects the source–sink balance. The plant parts that form the sink can, therefore, be markedly different between crop species.

For maize, the sink strength of the harvestable parts constitutes the developing ears, which can be seen as a collection of competing kernels. Sink strength at the plant level is largely controlled by the crop growth rate around silking, which strongly determines the number of kernels that set and fill (Tollenaar *et al.*, 1992; Vega *et al.*, 2001). The utilization of hybrid technology in maize has led to increased light capture and utilization via enhanced growing season length, and increased leaf area index and stay-green traits to maintain photosynthetic efficiency longer, jointly raising source capacity by an estimated 113% (Lee and Tollenaar, 2007). Since the harvest index in hybrids is maintained at ~50%, sink strength appears to have increased in proportion to source capacity, which can be explained by the strong relationship between dry matter accumulation around silking and establishment of sink size via kernel number (Echarte *et al.*, 2004). Further evidence for the role of photosynthesis in seed set and grain filling comes from defoliation experiments. Defoliation in maize leads to a decrease in grain yield (Barnett and Pearce, 1983) and grain quality (Shekoofa *et al.*, 2010), with the effect on kernel number and grain filling being dependent on the timing of defoliation. Leaf removal around silking has a strong impact on kernel number and yield, whereas leaf removal at later stages only impacts grain filling and has less impact on yield (Tollenaar and Daynard, 1978).

In sugarcane, the sink constitutes the sugar-accumulating culms, composed of elongated internodes. The accumulation of sugar to high concentrations (~500 mM sucrose in internode juice; Wu and Birch, 2006) is facilitated by several specialized features in the sucrose loading and translocation pathway (Wang *et al.*, 2013). It includes high expression of *SWEET13* sugar transporter genes in the photosynthetically mature leaf parts (Hu *et al.*, 2018), a fine balance between soluble acid invertase and sucrose phosphate synthase to control the rate of sucrose formation in internodes, and expression of a specific sucrose transporter gene *ShSUT1* to prevent sucrose backflow into the apoplast (Rae *et al.*, 2005). Despite these specialized features, accumulation of sucrose in leaves of sugarcane can have a strong negative feedback on photosynthetic capacity. Partial shading or intermittent darkening of leaves alleviates feedback inhibition of photosynthesis (McCormick *et al.*, 2006, 2008*a*; Ribeiro *et al.*, 2017), whereas cold girdling or exogenous sucrose application promotes down-regulation of photosynthesis (McCormick *et al.*, 2008*b*; Lobo *et al.*, 2015). The inhibiting effect of sucrose accumulation seems to rely on signals derived from the concomitant accumulation of hexoses, trehalose-6-phosphate (T6P), and/or expression of hexokinase. The manipulation of these signals may allow decoupling of source activity from sink feedback and enable even greater sucrose accumulation (McCormick *et al.*, 2009; Chandra *et al.*, 2011).

In sorghum, the parts constituting the relevant sinks for yield are dependent on the variety, and can vary from the grains (grain sorghum) to the stem (sweet sorghum), or a combination of both in dual-purpose production systems. In sweet sorghum, the accumulation of sucrose in the stem internodes is facilitated by altered expression of several sucrose transporters (Milne *et al.*, 2013) and vacuolar invertase isoforms (Chi *et al.*, 2020), compared with grain varieties. In grain sorghum, grain sink strength is strongly determined by seed set in the panicle, which in turn depends on the crop growth rate around anthesis. As a result, photosynthetic activity around this period is especially important for sink formation and yield, similar to the situation in maize. Consistently, leaf removal at booting and anthesis stages has a strong negative impact on grain sorghum yield via reduction in seed number, as well as average seed weight (Stickler and Pauli, 1961; Legwaila *et al.*, 2013). In dual-purpose production systems, both stem and grain are important sinks for yield. While the elongating internodes are potent sinks during vegetative growth, the grains develop later. Panicle pruning in a range of tropical sorghum genotypes did not affect stem sugar content (Gutjahr *et al.*, 2013*a*, *b*), suggesting that competition between both sinks is largely prevented due to the temporal separation in development.

Crop management can have important implications for source–sink interactions. Strong source activity during the development and filling of sinks is needed for stable and high yields, but can be affected by the timing of planting. For example, late planting in sub-Saharan Sudan–Sahelian climates can expose sorghum to severe post-anthesis droughts, which negatively impacts grain filling (Tovignan *et al.*, 2016). Longer maintenance of green leaf area during drought periods via stay-green traits can mitigate some of this yield loss in both grain and sweet sorghum (Borrell *et al.*, 2000; Tovignan *et al.*, 2016). Planting dates can also impact source–sink interactions at crucial developmental stages. For example, late plantings of summer maize to align crop growth with the timing of rainfall in rain-fed cropping systems of Argentina can push back the period of grain filling into weather with unfavourably low light levels, leading to low photosynthetic activity, decreased grain filling, and yield loss (Bonelli *et al.*, 2016). A similar situation occurs in temperate monsoon climates in Northern China, where late plantings can confine maize grain filling to a period with suboptimal light levels, in this case caused instead by the onset of the rainy season (Gao *et al.*, 2017).

### Increasing plant density leads to a lower source–sink ratio

A major trend in crop production systems of grain crops is the steady increase in plant density. In species with only a few tillers such as sorghum, or with only a single stem such as maize, increasing plant density will increase the number of ears or panicles per unit land area. Taking maize as an example, planting density across nine US corn belt states has increased by 0.07±0.01 plants m^−2^ year^−1^ since the 1990s (Assefa *et al.*, 2018) and is seen as an important factor underlying the steady increase in maize yield. How attainable these yield gains are depends on other crop management factors such as the fertilization level (Ruffo *et al.*, 2016), as well as the weather conditions during the growing season. Despite breeding efforts to facilitate high planting density, for example steeper leaf angles, under stressful conditions such as drought, increased plant density may enhance year-by-year yield variability, via increased competition between neighbouring plants (Lobell *et al.*, 2014).

**Fig. 6.**
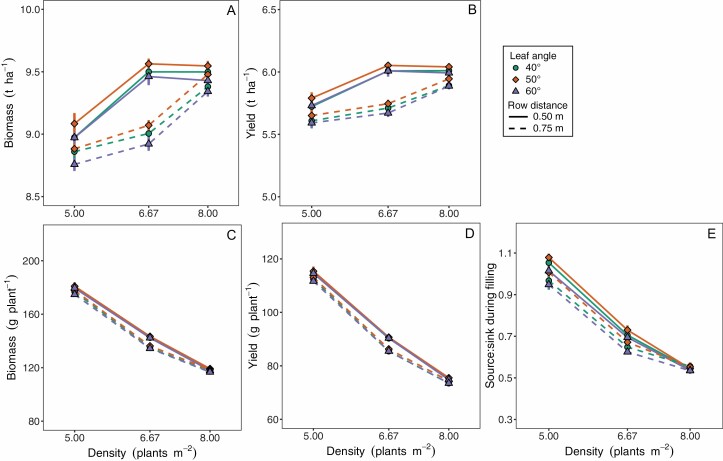
Simulated biomass productivity, yield and source-sink ratio of maize grown at different plant densities. Results are shown at field (A, B) and plant level (C, D) as means ±SD of 4–5 simulations. Source: sink ratio (E) was calculated during the grain-filling period. Input parameters were varied to simulate different leaf declination angles of 40°, 50°, and 60° (different symbols); and common row distances (Li *et al.*, 2015; USDA, 2020b) of 0.50 m (solid line) and 0.75 m (dotted line). For a full description of the model and parameters, see Evers and Bastiaans (2016).

In addition, we reason that the increases in planting density have profound effects on source–sink balance by promoting sink strength, whereas photosynthetic activity is largely determined by the incident irradiance and much less impacted by plant density. To demonstrate the impact of planting density on source–sink balance further, we used a 3D functional–structural model (previously described by Evers and Bastiaans, 2016) to simulate a maize crop at different planting densities. The model simulations show that for the observed increase in average maize plant density from five to eight plants m^−2^ between 1987 and 2016 (Assefa *et al.*, 2018), productivity and yield per unit area marginally increase (Fig. 6A, B), but productivity and yield per plant decline (Fig. 6C, D) while source–sink balance approximately halves (Fig. 6E). These results are only weakly affected by more upright leaf angles (different symbols, Fig. 6), which favours canopy photosynthetic activity in modern maize hybrids via more uniform vertical light distribution across the canopy (Ort *et al.*, 2015). Thus, crop yield becomes more strongly source limited with increasing plant density, and the general trend of increasing plant density is likely to enhance the importance of photosynthetic efficiency for yield, especially in the grain crops sorghum and maize.

## Conclusions

In this review we have explored the case for improvement of photosynthetic efficiency in C_4_ crops as a means to enhance productivity and yield. Despite the limited focus on improving photosynthetic efficiency in C_4_ compared with C_3_ species, there appears to be substantial evidence that this strategy may be achievable and beneficial for yield. Using model analysis and literature review, several tangible bottlenecks within the C_4_ pathway could be identified which exert strong control under relevant conditions for crop productivity, some of which can be alleviated via leveraging natural genetic variation in crop breeding programmes, whereas others may only be improved successfully via transgenic or gene editing methods. The decline in source–sink balance due to increases in planting density is likely to enhance the importance of photosynthetic efficiency for yield. Considering the predicted magnitude of the shortfall between food supply and demand, the timelines involved in crop breeding programmes, and the importance of C_4_ crops for global food, feed, and fuel production, implementing photosynthetic improvement as part of the C_4_ crop improvement toolbox is both urgent and timely.
